# Outcome of glioblastoma patients after intensive care unit admission with invasive mechanical ventilation: a multicenter analysis

**DOI:** 10.1007/s11060-023-04403-6

**Published:** 2023-08-02

**Authors:** Bernhard Neumann, Julia Onken, Nicole König, Henning Stetefeld, Sebastian Luger, Anna-Luisa Luger, Felix Schlachetzki, Ralf Linker, Peter Hau, Elisabeth Bumes

**Affiliations:** 1 Department of Neurology, Regensburg University Hospital, Regensburg, Germany; 2 Department of Neurology, Donau-Isar-Klinikum Deggendorf, Deggendorf, Germany; 3 Department of Neurosurgery, Charité-Universitätsmedizin Berlin, corporate member of Freie Universität Berlin, Humboldt-Universität Zu Berlin, Berlin, Germany; 4 Department of Neurology, Faculty of Medicine and University Hospital Cologne, University of Cologne, Cologne, Germany; 5 Department of Neurology, RKH Klinikum Ludwigsburg, Ludwigsburg, Germany; 6 Department of Neurology, Goethe University Frankfurt, University Hospital, Frankfurt am Main, Germany; 7 Dr. Senckenberg Institute of Neurooncology, Goethe University Frankfurt, University Hospital, Frankfurt am Main, Germany; 8 Frankfurt Cancer Institute (FCI), Goethe University Frankfurt, Frankfurt am Main, Germany; 9 University Cancer Center (UCT), Goethe University Frankfurt, University Hospital, Frankfurt am Main, Germany; 10 German Cancer Research Center (DKFZ) Heidelberg, Germany and German Cancer Consortium (DKTK), Partner Site Frankfurt/Mainz, Frankfurt am Main, Germany; 11 Wilhelm Sander-NeuroOncology Unit, Regensburg University Hospital, Regensburg, Germany

**Keywords:** Glioblastoma, Intensive care unit, Invasive mechanical ventilation, Outcome

## Abstract

**Purpose:**

Patients with glioblastoma are exposed to severe symptoms and organs failures (e.g., coma or acute respiratory failure), that may require intensive care unit (ICU) admission and invasive mechanical ventilation (IMV). However, only limited data are available concerning the prognosis of patients with glioblastoma receiving IMV. We sought to describe the reasons for ICU admission, and outcomes of patients with glioblastoma requiring IMV for unplanned critical complications.

**Methods:**

In this retrospective analysis, four certified interdisciplinary brain tumor centers performed a retrospective review of their electronic data systems. All patients with glioblastoma admitted to an in-house ICU and receiving IMV between January 2015 and December 2019 were included. Clinical and prognostic factors as well as relevant outcome parameters were evaluated by group comparisons and Kaplan Meier survival curves.

**Results:**

We identified 33 glioblastoma patients with a duration of IMV of 9.2 ± 9.4 days. Main reasons for ICU admission were infection (n = 12; 34.3%) including 3 cases of Pneumocystis jirovecii pneumonia, status epilepticus (31.4%) and elevated intracranial pressure (22.9%). In-hospital mortality reached 60.6%. Younger age, low number of IMV days, better Karnofsky Performance Status Scale before admission and elevated intracranial pressure as cause of ICU admission were associated with positive prognostic outcome.

**Conclusion:**

We conclude that less than 50% of patients with glioblastoma have a favorable short-term outcome when unplanned ICU treatment with IMV is required. Our data mandate a careful therapy guidance and frequent reassessment of goals during ICU stay.

## Introduction

Glioblastoma is the most common primary malignant brain tumor [1] with a median overall survival ranging from 12 to 18 months [2] despite multimodal therapy including surgery, radiotherapy, alkylating chemotherapies with temozolomide [3] or temozolomide / lomustine [4] and tumor treating fields [5]. The disease course itself, e.g., with developing brain edema resulting in elevated intracranial pressure or a structural epilepsy [6, 7] as well as complications derived from the tumor therapy, such as opportunistic infections, may require admission to an intensive care unit (ICU) [8].

Due to poor prognosis of the primary disease, therapy guidance and regular reassessment of treatment goals of glioblastoma patients in the ICU with invasive mechanical ventilation (IMV) is needed. Structured data on morbidity and mortality to guide decision-making are currently not available for glioblastoma patients. Recent publications often include various primary malignant brain tumors with a heterogenous prognosis and are retrospective by nature [9–11]. In these publications, the proportion of patients with glioblastoma was mostly below 50%, and less than 50% of brain tumor patients received IMV during ICU stay [9–11]. To the best of our knowledge, subgroup analyses of patients with glioblastoma who were invasive mechanically ventilated are missing in all publications.

Intriguingly, most patients with primary brain tumors admitted to an ICU presented at discharge from hospital with stable or improved functional status as assessed by the Karnofsky Performance Status Scale (KPS) [9, 11], an observation that could be related to a high remission rate of comorbidities causing ICU admission, like infections or seizures. In accordance with this hypothesis, the most common reasons for ICU admission of patients with primary brain tumors were respiratory failure, septic shock, refractory epileptic seizures, and non-epileptic coma [9–11].

In contrast, published data on metastatic non-primary brain tumors show that diagnosis of metastasis may prevent ICU admission [12], even when potentially reversible reasons as infections or seizures are present. Therefore, it is important to keep in mind that the ICU mortality rate of patients with cancer is comparable to patients with non-oncological diseases [13–15] and should therefore not cause ICU refusal.

The primary objective of our retrospective multicenter study was to assess the in-hospital mortality of patients with glioblastoma who were admitted to an ICU and required IMV. In addition, the reasons for ICU admission and survival after discharge were analyzed.

## Methods

### Study design and patient selection

Four certified interdisciplinary brain tumor centers with access to a specialized in-house ICU (Regensburg University Hospital, Charité - Universitätsmedizin Berlin, University Hospital Cologne, University Hospital Frankfurt; Germany) took part in the study. All consecutive patients between 2015 and 2019 were included if they had histologically proven glioblastoma (according to the “WHO Classification of Tumors of the Central Nervous System” in the respective valid version at the time of diagnosis) and required IMV. Non-invasive mechanical ventilation was not sufficient for inclusion. IMV was performed due to coma, elevated intracranial pressure, sepsis with respiratory failure, airway protection or refractory status epilepticus. Other inclusion criteria were at least 18 years of age and an acute medical condition needing ICU treatment and IMV. Patients with elective tumor surgery, who needed transient ventilation during and after surgery, were excluded. All patients were treated according to national and international guidelines in interdisciplinary teams including experienced neuro-intensive care physicians and neuro-oncologists. To assess our outcome parameters, institutional databases and medical records were checked with a cut-off date of 1st November 2021.

### Outcome parameters

Data on baseline demographics, clinical information about course of disease and treatment of glioblastoma until admission to ICU, reason for ICU admission, clinical course on ICU and outcome after discharge including placement, date of death, and reason for death were obtained. In addition, KPS, tumor therapy and some laboratory values were recorded. The following definitions were used to categorize the reasons for ICU admission: The diagnosis of elevated intracranial pressure was made by experienced intensive care physicians and neuro-oncologists based on clinical and imaging parameters and where available also intracranial pressure measurements. Seizures were defined clinically (mainly motoric) or according to EEG activity. The diagnosis of status epilepticus was made according to national guidelines based on clinical parameters and EEG findings.

### Statistics

GraphPad Prism 5® (GraphPad Software, La Jolla, USA) was used for statistical analysis. Data were presented as mean with standard deviation, median and range (as indicated) or total number with percentage. Group-comparison was tested with either Mann-Whitney U test or Fisher’s exact test. The both-sided significance level was set to α = 0.05. Survival after ICU discharge is shown as Kaplan Meier survival curve.

## Results

### Characteristics of the study group

We identified 33 patients with glioblastoma who fulfilled all inclusion criteria. Mean age at admission to ICU was 60.3 ± 14.1 years (median 62, range 32–78), and 33% of the patients were female (Table 1). Glioblastoma was diagnosed 170.6 ± 214.9 days (median 67, range 0–799) before admission to ICU. In seven patients, the histological diagnosis of glioblastoma was made during ICU stay. 13 patients had not yet started tumor-specific treatment at the time of ICU admission.

**Table 1:** Baseline characteristics and Outcome

Glioblastoma patients (n)	33
**Baseline characteristics**	
Male / Female (n)	22 / 11
Age (years)	60.3 ± 14.1 (62, 32–78)
KPS before ICU	75% ± 15 (80, 40–100)
IDH wild-type status available (n)	21 / 26 (80.8%; 7 unknown)
Days between first diagnosis and ICU	170.6 ± 214.9 (67, 0–799**)**
Glioblastoma specific therapy before ICU (n)	
No specific therapy	13 (39.4%)
First-line	10 (30.3%)
First relapse	6 (18.2%)
Second relapse	2 (6.1%)
Unknown	2 (6.1%)
Documented ACP existing (n)	8 / 32 (25%, 1 unknown)
Cause for ICU stay (n, %) *	
Infection	12 (34.3%)
Status epilepticus	11 (31.4%)
Elevated intracranial pressure	8 (22.9%)
Intracranial bleeding	1 (2.9%)
Stroke	1 (2.9%)
Pulmonary embolism	1 (2.9%)
Unexplained asystole	1 (2.9%)
**Outcome**	
Invasive mechanical ventilation (days)	9.2 ± 9.4 (6, 1–41)
ICU (days)	11.6 ± 9.8 (8, 1–41)
In-hospital mortality (n, %)	20 (60.6%)
Discharge to home (n, %)	6 (18.2%)
Discharge to rehabilitation or care facility (n, %)	7 (21.2%)
Cause of in-hospital mortality (n, %)	
Multiple organ failure / sepsis	10 (50%)
Early therapy limitation	4 (20%)
Superrefractory status epilepticus	3 (15%)
CPR unsuccessful	3 (15%)
Total therapy limitations (n, %)	14 of 20 deceased (70%)
Days between therapy limitation and admission	12.6 ± 9.9
Reason for therapy limitation	
According to the presumed patient´s will be stated by close relatives (legal representative)	12 (85.7%)
According to documented ACP	1 (7.1%)
According to patient´s will be stated by the patient	1 (7.1%)

“Age”, “KPS before ICU”, “Days between first diagnoses and ICU admission”, “Invasive mechanical ventilation (days)”, “ICU (days)” and “Days between therapy limitation and admission“ are depicted as mean **±** standard deviation as well as median and range, if applicable (in brackets), other parameters in total number with percentage (in brackets)

* In two cases two plausible causes for ICU stay were found so that n = 35 for “cause for ICU stay”

KPS: Karnofsky Performance Status Scale; ICU: intensive care unit; IDH: isocitrate dehydrogenase; CPR: cardiopulmonary resuscitation. ACP: advanced care planning

### Reasons for admission to ICU

The main reason for ICU admission was infection (n = 12; 34.3%), including three cases of Pneumocystis jirovecii pneumonia, two cases of intracerebral abscess, two cases of peritonitis because of sigma perforation, one case of post-operative meningitis, one case of Legionella pneumonia and three cases of pneumonia with unknown or common pathogens. Other frequent reasons were status epilepticus (31.4%) and elevated intracranial pressure (22.9%) (Table 1). All patients who were admitted for Pneumocystis jirovecii pneumonia had prophylactic antibiotic therapy with cotrimoxazole during radio-chemotherapy. In the group of 14 patients who were admitted to ICU within 40 days of glioblastoma diagnosis we found two patients with status epilepticus, one patient with subdural hygroma and one with hydrocephalus needing surgery, one with incidental stroke, one with Legionella pneumonia and one with unexplained asystole, as well as seven patients with first diagnosis of glioblastoma presenting in two cases with status epilepticus and in five cases with elevated intracranial pressure. Patients with newly diagnosed glioblastoma and treatment on ICU had significant more often elevated intracranial pressure compared to patients with glioblastoma who were admitted to ICU at a later stage of disease (71.4% vs. 11.5%; p = 0.004). Patients who were admitted within 40 days of glioblastoma diagnosis had a significantly lower frequency of infections as reason for ICU treatment (7.1% vs. 57.9%; p = 0.0036).

### Short-term outcomes

The duration of IMV was 9.2 ± 9.4 days (median 6, range 1–41). In-hospital mortality reached 60.6%, mainly due to sepsis and multi-organ failure (50%). In four cases (20%), ICU treatment was terminated early (within 6 days of admission) because of the diagnosis of glioblastoma and comorbidities. In 10 other cases, therapy limitations were implemented after intense treatment attempts and expected unfavorable outcome due to complications of glioblastoma. Therefore, in total, therapy limitations were set in 14 cases of patients not surviving ICU (70%) (Table 1). The decision for therapy limitation was made in average 12.6 ± 9.9 days (median 10, range 2–41) after admission. In 10 cases with intense therapy attempts, therapy limitation was decided after 15.3 ± 10.0 days (median 12, range 7–41) of treatment at ICU. Decisions were mainly made on presumed patient`s will after consultation of close relatives, because only 25% in our cohort had documented advanced care planning. In total, six patients (18.2%) could be discharged directly from hospital to their homes, six patients (18.2%) were discharged to a rehabilitation unit and one patient (3%) was discharged to a care facility.

### Prognostic factors of in-hospital mortality

We also investigated prognostic factors that influenced in-hospital mortality. Patients who recovered from their ICU stay were younger (52.8 ± 17.7 vs. 65.2 ± 8.7 years; p = 0.08), had a higher KPS at ICU admission (82 ± 16 vs. 72 ± 14; p = 0.08) and a shorter course of disease before ICU stay (138.9 ± 232.2 [median 157] vs. 191.2 ± 206.4 days [median 18]; p = 0.27) (Table 2). A KPS < 80% was associated with an increased probability to die at ICU (75% vs. 46.7%; p = 0.24), even if the risk was not significant.

**Table 2:** Characteristics of surviving and deceased patients (in-hospital mortality)

Patients	Deceased (n = 20)	Surviving (n = 13)	P-value
Age (years)	65.2 ± 8.7 (66, 50–75)	52.8 ± 17.7 (51, 32–78)	*0.08*
KPS before ICU	72 ± 14 (70, 40–90)	82 ± 16 (90, 50–100)	*0.08*
IDH wild-type	93.3%	63.6%	0.13
Days between first diagnosis and ICU admission	191.2 ± 206.4 (157, 0–799)	138.9 ± 232.2 (18, 0–799)	0.27
Tumor progression (last 2 months)	30.1%	35.0%	1
Cause of ICU admission			
Infection	10 (45.5%)	2 (15.4%)	0.14
Status epilepticus	7 (31.8%)	4 (30.8%)	1
Elevated intracranial pressure	2 (9.1%)	6 (46.2%)	*0.032*
Other	3 (13.6%)	1 (19.6%)	
Days of invasive mechanical ventilation	11.5 ± 9.4 (10.5, 1–41)	5.7 ± 8.8 (3, 1–34)	*0.008*

“Age”, “KPS before ICU” and “Days between first diagnoses and ICU admission” are depicted as mean **±** Standard Deviation as well as median and range (in brackets), other parameters in total number or / with percentage (in brackets). Mann-Whitney U test was used for age-differences, “KPS before ICU”, “day between first diagnoses and ICU admission” and “days of invasive mechanical ventilation”. For other parameters Fisher`s exact test was used

KPS: Karnofsky Performance Status Scale; ICU: intensive care unit; IDH: isocitrate dehydrogenase

The in-hospital mortality in the group of patients with diagnosis made at ICU (n = 7) was lower than in the total cohort (42.9% vs. 60.6%; p = 0.43). In contrast, patients who have already had at least one relapse deceased in 83.3% during their ICU stay (p = 0.27 for first diagnosis vs. tumor relapse).

Irrespective of demographic factors, patients with elevated intracranial pressure as reason for ICU admission were more likely to have a favorable outcome (46.2% vs. 9.1%; p = 0.032). Patients with infections had a tendency for a less favorable outcome (45.5% vs. 15.4%; p = 0.14), especially if they suffered from opportunistic pathogens like Legionella pneumonia (n = 1), candida glabrata sepsis (n = 1) or Pneumocystis jirovecii pneumonia (n = 3). If status epilepticus was the reason of ICU admission, patients survived in about 50% of cases, but superrefractory status epilepticus (n = 3) did lead to death in all affected patients.

Surviving patients had a significantly lower number of days with IMV (5.7 ± 8.8 days vs. 11.5 ± 9.4 days; p = 0.008) and only one survivor needed IMV for more than eight days.

### Long-term follow-up of survivors

**Fig. 1:**
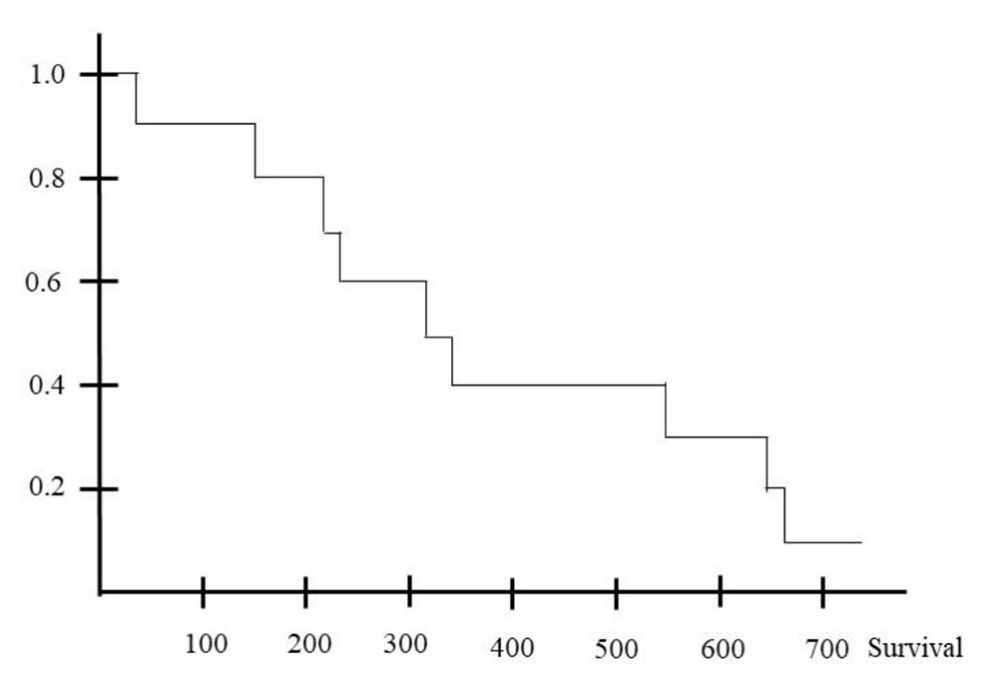
Survival after ICU discharge. Shown is the survival in days after ICU discharge of 10 patients with a maximum follow-up of 750 days as Kaplan-Meier survival curve

Three patients were lost for follow-up directly after discharge, so follow-up analysis was possible in 10 patients (76.9%) with a maximum of 750 days. Mean survival was 394.9 days ± 246.1 (median 340), with one patient being still alive 750 days after ICU discharge at last follow-up. 60% of surviving patient died within one year after ICU discharge (Fig. 1). Tumor progression was the leading cause of death in 60% of patients (6 out of 10); in three patients, the mechanism of death could not be identified.

## Discussion

To the best of our knowledge, we are the first who report on a homogeneous group of patients with glioblastoma and IMV after unplanned admission to ICU. We revealed infections and status epilepticus as major reasons for ICU admissions in patients with glioblastoma. Overall, in-hospital mortality was 60.6%. However, younger age, better KPS, and the admission reason “elevated intracranial pressure” were associated with a favorable prognosis.

In concordance with previous publications on patients with primary brain tumors [9–11] infectious complications and refractory seizures / epileptic status were common reasons for ICU admission in our cohort of glioblastoma patients.

The high rate of opportunistic infections (with e.g., Pneumocystis jirovecii and Legionella pneumoniae) leading to ICU admission is not surprising, as glioblastoma patients frequently receive chemotherapeutic agents and high doses of corticosteroids, which are both immunosuppressive [8]. Consistent with this, significantly less infectious complications occurred in the group of patients who were diagnosed within 40 days before ICU admission and therefore often had not yet started with chemotherapy.

Elevated intracranial pressure was a frequent reason for admission to ICU in our cohort and was seen particularly in patients whose initial diagnosis of glioblastoma was made during the ICU stay. In contrast to our data, elevated intracranial pressure has not been reported as independent reason for ICU admission in previous publications [9–11]. This is probably based on a lack of reporting elevated intracranial pressure in part of these publications. In this situation, decompressive neurosurgical resection provides acute relief and contributes to a better outcome. Of note, patients with glioblastoma developing intracranial pressure in the final stage of their tumor disease are commonly not treated at ICU, and of course in these patients elevated intracranial pressure presumably will not be associated with better outcome. The association between elevated intracranial pressure and relatively good outcome in our cohort is restricted to newly diagnosed glioblastoma patients.

Epileptic seizure is a common initial symptom of a brain tumor and more than half of patients with glioblastoma develop symptomatic epilepsy during the course of disease [16]. Therefore, it is not surprising that epileptic seizures are a frequent reason for emergency hospitalization of patients with brain tumors [17], as also confirmed in our analysis of admission reasons for ICU. The reason “epileptic seizure / status” takes on a special role, as it is a potentially reversible condition that is not primarily linked to organ dysfunction like e.g. sepsis. Nevertheless, in the presence of superrefractory status epilepticus, that does not respond to a variety of anticonvulsant medication, is associated with an unfavorable prognosis.

Remarkably, patients with primary brain tumors in previously published cohorts were less severely ill than patients in our cohort. In contrast to our study, only a subset of patients (< 50%) was invasively mechanically ventilated in other studies with primary brain tumors and the length of stay at ICU was shorter [9, 11]. This and other aspects as well as the selection for glioblastoma, which is connected with a specifically unfavorable prognosis, may contribute to the in-hospital mortality of 60,6% in our cohort, which is higher than reported in cohorts of primary brain tumor patients [10, 18] and solid cancer patients [13–15]. Furthermore, patients with glioblastoma experience not only complications of tumor disease and therapy but also neurological complications with unfavorable prognosis. Neurological complications often lead to admission to the ICU (e.g., status epilepticus) and are usually not expected in patients with solid tumors unless metastases to the central nervous system are present.

A high rate of treatment limitations likely also contributes to the increased in-hospital mortality reported in our cohort. In 14 of 20 patients deceased during ICU stay, treatment limitations were set due to unfavorable neuro-oncological prognosis. However, only 25% of patients had documented advanced care planning. This seems low in the context of a severe cancer diagnosis that should lead to early initiation of end-of-life decisions. However, similar rates have been reported in the literature [19] and may be based on e.g. avoidance behavior of patients, reluctance of physicians to communicate bad news, organizational problems or early-onset neurocognitive deficits [20]. A selection bias for the low number of patients with documented advanced care planning can be discussed, as patients who already made decisions on different scenarios at the end of their disease probably refused ICU admission or IMV.

Assessment of prognosis on brain tumor patients typically includes patient-intrinsic factors (e.g. age, KPS and comorbidities), disease-intrinsic factors (e.g. histology, molecular pattern, localization and symptom load) and treatment-intrinsic factors (e.g. extent of resection, ability to receive full treatment, acute reason for admission, organ function and catecholamine demand) [21, 22]. However, there is no established score for prognostic assessment in oncological patients hospitalized in an ICU. Therefore, prognosis and decisions on treatment termination should be evaluated regularly with regard to the patient’s will and by a multi-professional team including the ICU team, neuro-oncologists / oncologists and, if indicated, palliative care physicians [22]. As patients reported here were treated within certified interdisciplinary brain tumor centers with access to an in-house ICU, involvement of intensive care physicians and neuro-oncologists / neurosurgeons in the decision-making process was given. The decision to withdraw treatment was made on average 12.6 days after admission to the ICU, indicating a serious treatment attempt and careful consideration of options.

Approximately half of the survivors in follow-up lived longer than one year after ICU discharge, which is a considerable high survivor rate also in view of other publications [18]. As expected, glioblastoma progression was the main reason for death in our cohort of survivors. However, the long post-ICU survival of some patients despite their diagnosis of glioblastoma could be due to a positive and meaningful selection of patients who were treated with maximal measures. We speculate that the collective experience of neuro-oncologists / neurosurgeons and specialized ICU´s in dedicated brain tumor centers contributed to these favorable outcomes. Importantly, not only the length of survival but also the functional status and the health-related quality of life are of great relevance for the survivors. However, due to incomplete follow up and the retrospective design, we are not able to describe the functional status of patients after discharge.

There are some weaknesses of the presented study. Despite the multicenter approach, it was not possible to include a larger cohort of patients, which was due to strict inclusion criteria. In addition, the low number of cases suggests, that the hurdle to indicate ICU therapy in patients with glioblastoma is probably high. Some patients with glioblastoma may have ruled out treatment in an intensive care unit considering the incurable tumor disease. Due to the retrospective design, no conclusions can be drawn regarding functional status after ICU discharge or content of therapy limitation.

However, our study has also a major advantage. We present, for the first time, a homogeneous patient group with glioblastoma and IMV at ICU, for which no comparable data are available in the literature.

In conclusion, our results support a treatment attempt at a specialized ICU if the reason for ICU admission is potentially reversible, as a fraction of patients with glioblastoma survive for more than one year after discharge from ICU. Reversible reasons for ICU admission are mainly intracranial pressure, epileptic seizures and infections. Our data further mandate the importance of a multi-professional approach including neuro-oncologists / neurosurgeons and intensive care physicians for treatment stratification in patients with glioblastoma and IMV in an ICU situation.

## Data Availability

The datasets analyzed during the current study are available from the corresponding author on reasonable request.
